# A prediction model for urological tumor metastasis using liquid biopsy-derived biomarkers

**DOI:** 10.3389/fmed.2026.1718624

**Published:** 2026-07-03

**Authors:** Jiandong Qu, Jing Zhang, Xiaoli Huang

**Affiliations:** Department of Urology, Qingdao Municipal Hospital, Qingdao, Shandong, China

**Keywords:** inflammation-related parameters, liquid biopsy, metastasis, predictive indicators, random forest model, urological tumors

## Abstract

**Objective:**

To construct and validate a prediction model for tumor metastasis in patients with urological tumors based on liquid biopsy biomarkers and clinical characteristics, to facilitate early clinical identification of metastasis risk and formulation of individualized diagnosis and treatment plans.

**Methods:**

A total of 360 patients with urological tumors admitted to our hospital from January 2021 to December 2024 were retrospectively included. They were randomly divided into a training set (*n* = 252) and a validation set (*n* = 108) at a ratio of 7:3. Demographic characteristics and liquid biopsy biomarker indicators of the patients were collected. In the training set, demographic characteristics (age, gender) and liquid biopsy biomarkers (C-reactive protein, neutrophil count, monocyte count, platelet count, mean platelet volume, platelet distribution width, large platelet percentage, hemoglobin, white blood cell count, and urine parameters) were assessed. Univariate analysis was used to screen metastasis-related indicators, followed by Least Absolute Shrinkage and Selection Operator (LASSO) regression and multivariate Logistic regression to identify independent predictors. Three machine learning models (random forest, support vector machine, gradient boosting) were then constructed. The efficacy of the models was evaluated by the area under the receiver operating characteristic curve (AUC). The optimal model was selected, and the importance of key prediction indicators was analyzed.

**Results:**

There was no significant difference in baseline data between the training set and the validation set (*P* > 0.05). Multivariate Logistic regression indicated that C-reactive protein, neutrophil count, platelet count, platelet distribution width, hemoglobin, white blood cell count, and mean platelet volume were independent influencing factors for tumor metastasis in patients with urological tumors (all *P* < 0.05). The AUC of the random forest model (0.891) was significantly higher than that of the support vector machine (0.885) and the gradient boosting model (0.739), making it the optimal model.

**Conclusion:**

The random forest model constructed based on liquid biopsy predictive indicators can effectively predict tumor metastasis in patients with urological tumors. Neutrophil count, platelet count, and white blood cell count are key prediction indicators for urological tumor metastasis.

## Introduction

Urological tumors, including renal cancer, bladder cancer, prostate cancer, etc., have shown an increasing incidence year by year, seriously threatening the lives and health of patients (1). Tumor metastasis is the main cause of treatment failure and patient death. Early and accurate prediction of metastasis risk is crucial for improving prognosis (2). In recent years, liquid biopsy technology has received extensive attention in the field of tumor metastasis prediction due to its advantages such as minimally invasiveness and reproducibility (3). Studies have shown that the inflammatory response is closely related to tumor metastasis. Inflammatory indicators such as C-reactive protein and neutrophils can participate in the metastasis process by promoting the formation of the tumor microenvironment and enhancing the invasive ability of tumor cells. Meanwhile, platelet-related indicators (such as platelet count and platelet distribution width) play important roles in tumor cell adhesion, circulation survival, and metastasis colonization (4, 5). However, the predictive efficacy of a single biomarker is limited. Integrating multi-dimensional indicators to construct a prediction model is an important way to improve prediction accuracy (6). Machine learning algorithms can efficiently integrate multi - source data and have shown good performance in disease prognosis prediction. This study intends to combine liquid biopsy biomarkers and clinical characteristics to construct a prediction model for urological tumor metastasis through machine learning methods, identify key influencing factors, and provide an objective basis for early clinical intervention (7).

## Materials and methods

### Study subjects

A total of 360 patients with urinary system tumors who were hospitalized in our hospital from January 2021 to December 2024 were included in this retrospective study. They were randomly divided into a training set (*n* = 252) and a validation set (*n* = 108) at a ratio of 7:3. Inclusion criteria: (1) Aged 18–80 years; (2) Conforming to the above diagnostic criteria for urinary system tumors; (3) Complete clinical data and liquid biopsy test data; (4) Informed consent signed by the patients or their family members. Exclusion criteria: (1) Complicated with malignant tumors of other systems (such as lung cancer, gastric cancer, etc.); (2) A history of tumor metastasis or having received treatment for metastatic lesions (such as targeted therapy, radiotherapy) before enrollment; (3) Severe hepatic and renal insufficiency (serum creatinine > 178 μmol/L, Child-Pugh class C), coagulation dysfunction (international normalized ratio INR > 1.5); (4) Missing clinical data or follow-up data; (5) Lost to follow-up or withdrew from the study midway.

### Sample size calculation

In this study, sample size calculation was first carried out. Based on the expected incidence of the primary outcome indicator (urological tumor metastasis) (8), combined with previous relevant literature reports, preliminary pre-test results, and the clinical characteristics of the target population, the incidence was set at 20–22% (consistent with the actual metastasis incidence of this type of tumor), and a scheme compatible with the core statistical method (multivariate Logistic regression) was adopted. The calculation was completed through the “Sample Size Calculation” module of SPSS 26.0 and verified using the “pwr” package of R 4.2.1. The significance level was set at α = 0.05 (two-tailed), the test power at 1-β = 80%, and a 10% loss to follow-up rate was considered. Finally, the minimum required sample size was determined to be 320 cases. Accordingly, 360 patients with urological tumors from the Department of Urology of our hospital (from January 2020 to December 2024) were included. Among them, 252 cases were used as the training set and 108 cases as the validation set. The actual total sample size exceeded the minimum requirement, and its statistical power (1-β > 80%) was verified by SPSS 26.0, which could meet the statistical requirements of multivariate analysis.

### Data collection

The following information was collected through the electronic medical record system and laboratory database:

Demographic characteristics: age, gender.

Liquid biopsy biomarkers: C-reactive protein (CRP, mg/L), neutrophil count ( × 10^9^/L), neutrophil percentage (%), monocyte count ( × 10^9^/L), platelet count (PLT, × 10^9^/L), mean platelet volume (MPV, fL), platelet distribution width (PDW, %), large platelet percentage (P-LCR, %), hemoglobin (g/L), urine occult blood (positive/negative), red blood cell count ( × 10^12^/L), white blood cell count ( × 10^9^/L), squamous epithelial cells (cells/μL), non-squamous epithelial cells (cells/μL), urine glucose (positive/negative), urine specific gravity, bacterial count (cells/μL), hyaline casts (cells/μL), unclassified crystals (cells/μL).

### Outcome definition

With reference to the “Response Evaluation Criteria in Solid Tumors (RECIST 1.1)” (9) and the diagnostic consensus on urological tumor metastasis, tumor metastasis was defined as the presence of distant organ (such as lung, liver, bone, brain, etc.) or regional lymph node metastasis confirmed by imaging examinations (CT, MRI, bone scan, etc.) or pathological examinations. According to whether metastasis occurred, patients were divided into the metastasis group and the non-metastasis group.

### Statistical analysis

Statistical analysis was performed using SPSS 26.0 software and R software (version 3.4.2) and Python 3.9 software. Measurement data conforming to the normal distribution were expressed as mean ± standard deviation ($\bar{x}$ ± s), and independent-samples *t*-test was used for comparison between groups; non-normally distributed data were expressed as median (interquartile range) [M (Q1, Q3)], and Mann-Whitney U test was used for comparison between groups. Count data were expressed as number of cases (percentage) [n (%)], and the χ^2^ test or Fisher’s exact probability method was used for comparison between groups. In the training set, indicators related to tumor metastasis were screened by univariate analysis. After variable compression by Least Absolute Shrinkage and Selection Operator (LASSO) regression, independent influencing factors were determined by multivariate Logistic regression, and their odds ratios (OR) and 95% confidence intervals (CI) were calculated. Based on the independent predictors identified in the multivariate analysis, three machine learning prediction models, including random forest, support vector machine, and gradient boosting models, were constructed using the scikit-learn library in Python. To ensure model reproducibility and facilitate external validation, we explicitly defined the parameters for each of the three machine learning models. For the random forest model, the parameters were set as follows: number of trees = 500, maximum depth = 10, minimum samples split = 5, and random seed = 42. The support vector machine model employed a radial basis function kernel with a regularization parameter C = 1.0, gamma = “scale,” and random seed = 42. The gradient boosting model was configured with 100 boosting stages, a learning rate = 0.1, a maximum depth = 3, and random seed = 42. All continuous features were normalized using Z-score standardization based on the training set mean and standard deviation. The area under the receiver operating characteristic (ROC) curve (AUC) and 95% CI were used to evaluate the prediction accuracy. Following the classic principle of “at least 5–10 events per variable (EPV)”, this study preset that 10 prediction variables should correspond to at least 50 events, and the low correlation between variables was verified by the variance inflation factor (VIF < 2) to ensure the stability of the model. The significance level was set at α = 0.05.

## Results

### Comparison of general data between patients in the training set and the validation set

Among the 252 patients with urological tumors in the training set, 76 cases (30.16%) had tumor metastasis and 176 cases (69.84%) did not. Among the 108 patients with urological tumors in the validation set, 32 cases (29.63%) had tumor metastasis and 76 cases (70.37%) did not. There were no statistically significant differences between the two groups of patients in baseline data such as age and gender, liquid biopsy predictive indicators such as CRP, neutrophil count, and platelet count, urine test indicators such as urine occult blood and bacterial count, and the incidence of mild comorbidities (*P* > 0.05) (Table 1). The distribution of urological tumor types in the final 346 study subjects was as follows: 86 cases of renal cancer (24.86%), 168 cases of bladder cancer (48.55%), 92 cases of prostate cancer (26.59%), and there was no statistically significant difference in the tumor type distribution between the training set and the validation set (*P* > 0.05). In addition, the homogeneity test of mild comorbidities and tumor type distribution between the metastasis group and non-metastasis group in the total study population also showed no statistically significant difference (P > 0.05), indicating that mild comorbidities and tumor type distribution did not affect the research results (Table 2).

**TABLE 1:** Comparison of general data between patients in the training set and the validation set.

Indicators	Training set (*n* = 252)	Validation set (*n* = 108)	*t/χ^2^*	*P*
Age (years)	62.35 ± 9.12	63.17 ± 8.76	0.782	0.435
Gender [n (%)]			0.012	0.913
Male	168 (66.67)	72 (66.67)		
Female	84 (33.33)	36 (33.33)		
CRP(mg/L)	7.82 ± 3.25	8.15 ± 2.97	0.926	0.355
Neutrophil count ( × 10^9^/L)	5.68 ± 1.73	5.82 ± 1.65	0.674	0.501
Neutrophil percentage (%)	58.36 ± 8.42	59.12 ± 7.95	0.813	0.417
Monocyte count ( × 10^9^/L)	0.62 ± 0.21	0.59 ± 0.19	1.245	0.214
PLT ( × 10^9^/L)	235.67 ± 30.41	241.32 ± 31.78	1.593	0.112
MPV (fL)	9.85 ± 1.32	9.72 ± 1.25	0.870	0.385
PDW(%)	16.23 ± 2.15	15.98 ± 2.07	1.032	0.302
P-LCR(%)	28.65 ± 6.32	29.17 ± 5.98	0.741	0.459
Hemoglobin (g/L)	132.54 ± 18.67	130.89 ± 17.92	0.836	0.403
Urinary occult blood [n (%)]			0.087	0.768
Positive	102 (40.48)	43 (39.81)		
Negative	150 (59.52)	65 (60.19)		
Red blood cell count ( × 10^12^/L)	4.62 ± 0.58	4.57 ± 0.61	0.724	0.469
White blood cell count ( × 10^9^/L)	6.85 ± 2.13	7.02 ± 1.98	0.736	0.462
Squamous epithelial cells (cells/μL)	8.62 ± 3.15	9.03 ± 2.97	1.125	0.261
Non-squamous epithelial cells (cells/μL)	5.36 ± 2.08	5.12 ± 1.93	0.982	0.818
Urinary glucose [n (%)]			0.053	0.818
Positive	18 (7.14)	8 (7.41)	1.452	0.147
Negative	234 (92.86)	100 (92.59)		
Urinary specific gravity	1.015 ± 0.006	1.016 ± 0.005		
Bacterial count (cells/μL)	125.36 ± 49.42	132.67 ± 45.73	1.315	0.189
Hyaline casts (cells/μL)	0.32 ± 0.18	0.29 ± 0.15	1.367	0.172
Unclassified crystals (cells/μL)	2.15 ± 1.32	2.31 ± 1.25	0.921	0.357
Hypertension [n (%)]	98 (38.89)	40 (37.04)	0.110	0.740
Diabetes [n (%)]	56 (22.22)	23 (21.30)	0.038	0.845
Coronary heart disease [n (%)]	28 (11.11)	11 (10.19)	0.070	0.791
Chronic pulmonary disease [n (%)]	16 (6.35)	7 (6.48)	0.002	0.962
Tumor type [n (%)]			2.106	0.551
Renal cancer	60 (23.81)	26 (24.07)		
Bladder cancer	122 (48.41)	50 (46.30)		
Prostate cancer	64 (25.40)	28 (25.93)		
Other types^1^	6 (2.38)	4 (3.70)		

**TABLE 2:** Univariate analysis of influencing factors for tumor metastasis in patients with urological tumors.

Indicators	Metastasis group (*n* = 76)	Non-metastasis group (*n* = 176)	*t*/χ^2^	*P*
Age (years)	63.34 ± 9.22	62.01 ± 9.02	1.067	0.287
Gender [n (%)]			0.916	0.339
Male	50 (65.79)	118 (67.05)		
Female	26 (34.21)	58 (32.95)		
CRP(mg/L)	8.72 ± 3.33	7.42 ± 3.21	2.917	0.003
Neutrophil count ( × 10^9^/L)	6.77 ± 1.97	5.45 ± 1.52	5.766	0.001
Neutrophil percentage (%)	58.46 ± 8.52	58.11 ± 8.23	0.307	0.759
Monocyte count ( × 10^9^/L)	0.63 ± 0.25	0.59 ± 0.19	1.389	0.166
PLT ( × 10^9^/L)	249.69 ± 20.40	234.67 ± 15.01	6.891	0.001
MPV (fL)	10.99 ± 1.33	9.65 ± 1.30	1.670	0.001
PDW(%)	16.93 ± 2.25	16.13 ± 2.11	2.707	0.007
P-LCR(%)	28.75 ± 6.42	28.25 ± 6.02	0.593	0.554
Hemoglobin (g/L)	131.00 ± 18.31	137.74 ± 18.69	2.643	0.009
Urinary occult blood [n (%)]			0.106	0.744
Positive	32 (42.11)	70 (39.77)		
Negative	44 (57.89)	106 (60.23)		
Red blood cell count ( × 10^12^/L)	4.70 ± 0.68	4.60 ± 0.58	1.191	0.235
White blood cell count ( × 10^9^/L)	7.85 ± 2.65	6.65 ± 2.03	3.913	0.001
Squamous epithelial cells (cells/μL)	8.82 ± 3.24	8.58 ± 3.02	0.566	0.572
Non - squamous epithelial cells (cells/μL)	5.44 ± 2.42	5.26 ± 2.01	0.612	0.541
Urinary glucose [n (%)]			0.298	0.585
Positive	6 (7.89)	12 (6.82)		
Negative	70 (92.11)	164 (93.18)		
Urinary specific gravity	1.016 ± 0.008	1.015 ± 0.004	1.321	0.188
Bacterial count (cells/μL)	126.30 ± 49.33	124.05 ± 48.12	0.399	0.690
Hyaline casts (cells/μL)	0.33 ± 0.19	0.30 ± 0.11	1.573	0.117
Unclassified crystals (cells/μL)	2.19 ± 1.36	2.11 ± 1.29	0.444	0.657
Hypertension [n (%)]	30 (39.47)	68 (38.64)	0.015	0.902
Diabetes [n (%)]	18 (23.68)	38 (21.59)	0.132	0.716
Coronary heart disease [n (%)]	9 (11.84)	19 (10.80)	0.059	0.808
Chronic pulmonary disease [n (%)]	5 (6.58)	11 (6.25)	0.010	0.922
Tumor type [n (%)]			0.978	0.807
Renal cancer	18 (23.68)	42 (23.86)		
Bladder cancer	37 (48.68)	85 (48.30)		
Prostate cancer	19 (25.00)	45 (25.57)		
Other types^1^	2 (2.63)	4 (2.27)		

### Univariate analysis of influencing factors for tumor metastasis in patients with urological tumors

Univariate analysis showed that there were statistically significant differences in C-reactive protein, neutrophil count, platelet count, mean platelet volume, platelet distribution width, hemoglobin, and white blood cell count between the metastasis group and the non-metastasis group (*P* < 0.05) (Table 2).

### Multivariate Logistic regression analysis of influencing factors for tumor metastasis in patients with urological tumors

Whether urological tumor metastasis occurred was used as the dependent variable (1 = metastasis occurred, 0 = no metastasis occurred). The indicators with statistical significance in the univariate analysis were included in the LASSO regression for variable screening (Supplementary Table 1). Variables were selected using the screening criterion of lamda.1se (Supplementary Figures 1, 2). The indicators with a more appropriate number of predictive variables were included in the multivariate Logistic regression analysis. The results showed that CRP, neutrophil count, PLT, PDW, hemoglobin, white blood cell count, and MPV were independent influencing factors for tumor metastasis in patients with urological tumors (all *P* < 0.05) (Table 3).

**TABLE 3:** Multivariate Logistic regression analysis of influencing factors for metastasis status in patients with urological tumors.

Factor	β	SE	Wald	P	OR	95%CI
CRP	0.141	0.058	5.969	0.015	1.151	1.028–1.289
Neutrophil count	0.499	0.121	17.135	0.001	1.648	1.301–2.087
PLT	0.052	0.012	20.171	0.001	1.053	1.030–1.078
PDW	0.239	0.097	6.100	0.014	1.269	1.050–1.534
Hemoglobin	0.023	0.010	5.070	0.024	0.977	0.957–0.997
White blood cell count	0.272	0.082	10.873	0.001	1.312	1.117–1.542
MPV	0.686	0.153	20.039	0.001	1.985	1.470–2.680
Constant	−27.423	4.489	37.316	0.001	0.001	

### Prediction performance of machine learning models in the training set and validation set

**FIGURE 1:**
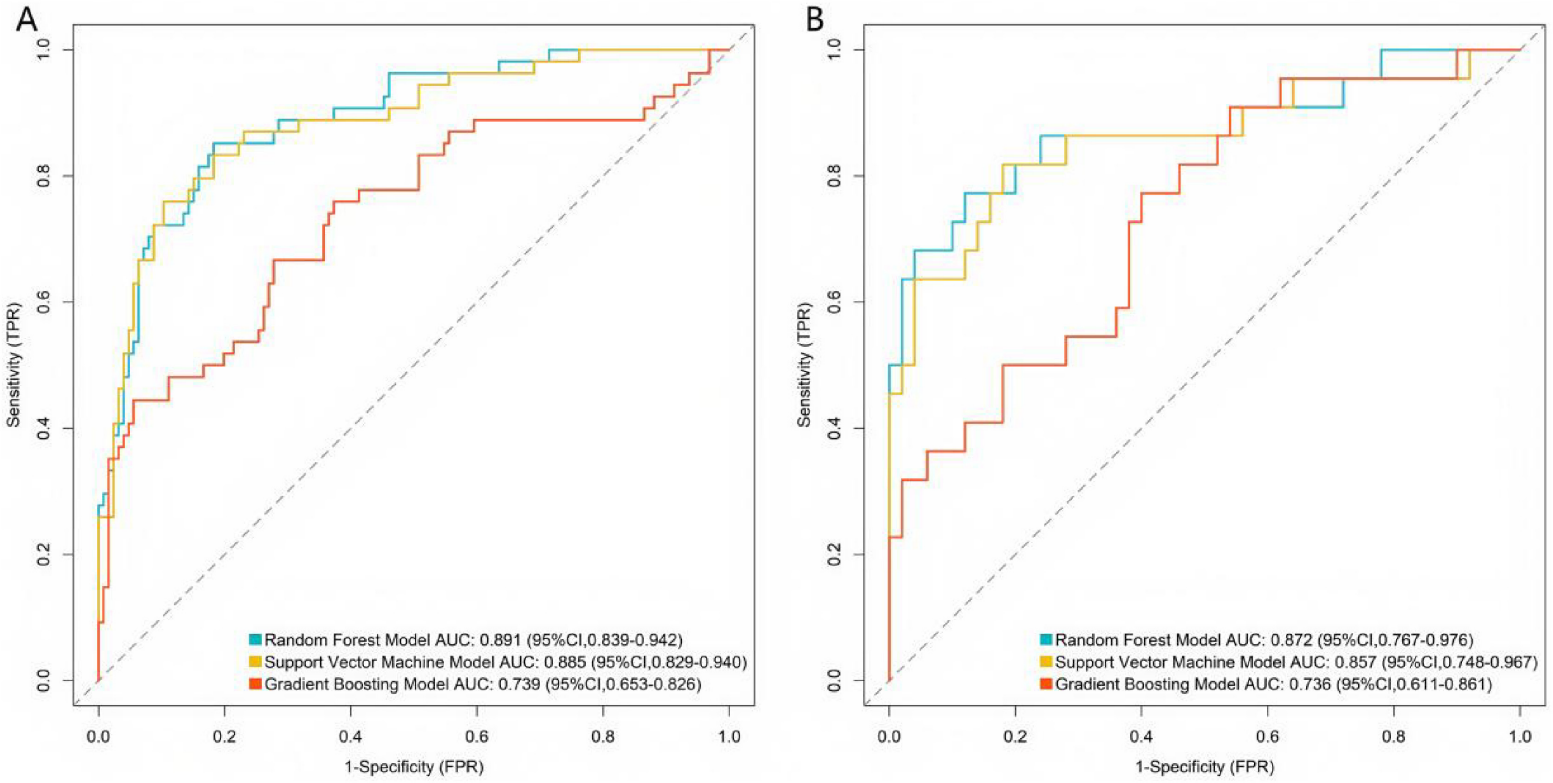
Area under the receiver operating characteristic curve of machine learning models. **(A)** The training set. **(B)** Validation set.

In the training set, the AUC values of the three models in the training set were 0.891 (random forest), 0.885 (support vector machine) and 0.739 (gradient boosting), respectively, and the corresponding AUC values in the validation set were 0.872, 0.857 and 0.736. The random forest model had the highest AUC value, and its AUC value in the validation set was highly consistent with that in the training set with a small decline range (Figure 1). Combined with the comprehensive evaluation of three aspects: the anti-overfitting stability of the model, the interpretability of feature importance, and the adaptability to clinical routine detection data, the random forest model was identified as the optimal prediction model for this study.

### Construction of a prediction model for urological tumor metastasis based on biopsy biomarkers

Based on the random forest model, the importance scores of independent influencing factors for the decline in the efficacy of urological tumor treatment were calculated. The importance ranking was as follows: PLT, neutrophil count, MPV, white blood cell count, PDW, hemoglobin, and CRP (Supplementary Figures 3, 4).

## Discussion

Urological tumor metastasis, as a core factor leading to treatment failure and patient death, its early and accurate prediction is crucial for improving prognosis (10, 11). In this study, based on the clinical data of 360 patients with urological tumors, liquid biopsy biomarkers were integrated. After variable screening by LASSO regression, seven independent risk factors were determined through multivariate Logistic regression, and a metastasis prediction model with the random forest model as the optimal one (AUC = 0.891) was constructed.

Among seven predictors, platelet count, neutrophil count, and white blood cell count ranked high in the feature importance ranking. We did not regard these single inflammation-related and platelet parameters as specific biomarkers for urological tumor metastasis, but integrated them to construct a combined prediction model by virtue of the random forest algorithm’s ability to capture the non-linear synergistic relationship between multiple indicators, which makes up for the lack of specificity of a single indicator. In addition, strict patient screening (excluding those with other systemic malignant tumors, severe inflammatory diseases, etc.) reduced the interference of non-tumor-related factors on indicator levels, further improving the specificity of the combined model for metastasis prediction. These parameters can thus serve as core predictive indicators for urological tumor metastasis. Although prospective validation is needed, they currently provide an objective basis for risk-stratified clinical decision-making, including preoperative planning of lymph node dissection extent, postoperative adjuvant therapy selection, and individualized surveillance schedules.

In this study, inflammation-related parameters served as key predictive indicators for urological tumor metastasis, and their predictive mechanism can be explained by multiple pathways through which the inflammatory microenvironment regulates the progression of urological tumors. These parameters are non-specific systemic indicators, and their effective application in this study relies on the strict exclusion of patients with other systemic malignant tumors and the characteristic combination pattern captured by the random forest model (12). Elevated neutrophil count, as an independent risk factor, may degrade the extracellular matrix by releasing matrix metalloproteinases, promoting tumor cell invasion and angiogenesis (13). Meanwhile, pro-inflammatory factors such as IL-8 and TNF-α secreted by neutrophils can activate the epithelial-mesenchymal transition (EMT) program of tumor cells, enhancing their metastatic ability. As a comprehensive indicator of the systemic inflammatory response, an elevated white blood cell count reflects the state of immune imbalance in the body-an increase in the infiltration of immunosuppressive cells such as tumor-associated macrophages can create conditions for tumor cell escape by inhibiting the function of cytotoxic T cells (14). C-reactive protein, as a classic inflammatory marker, its elevated level is closely related to the activation of the NF- κB pathway in the tumor microenvironment. This pathway can accelerate the formation of metastatic foci by upregulating the expression of pro-metastatic genes such as VEGF (15). These results are consistent with the theory of the “inflammation-tumor metastasis axis” in previous studies, confirming the central position of inflammatory-related parameters in the progression of urological tumors. For urological tumors, neutrophils can secrete higher levels of IL-8 in bladder cancer and prostate cancer tissues to further activate the EMT program of tumor cells, and the increased white blood cell count is more likely to induce immunosuppressive microenvironment in the pelvic cavity where urological tumors are located, which all promote tumor metastasis in a urological tumor-specific manner.

The independent predictive value of platelet-related indicators reveals the multiple mechanisms of action of platelets in tumor metastasis (15). An elevated platelet count can promote tumor cell survival by releasing platelet-derived growth factor (PDGF), and its surface adhesion molecules (such as P-selectin) can mediate the interaction between tumor cells and endothelial cells, facilitating the colonization of circulating tumor cells. PDW reflects the activation state of platelets, and its elevation indicates hyperactive platelet function, which may enhance the adhesion ability of tumor cells by releasing substances such as thromboxane A2. MPV, as a sensitive indicator of platelet activation, its increase is related to the release of pro-angiogenic factors (such as TGF-β) by platelets, which can accelerate the formation of the vascular network in metastatic foci. In this study, platelet count ranked first in feature importance, further supporting the key role of platelets as a “metastasis booster” in urological tumors. For example, platelet-derived growth factor (PDGF) can specifically accelerate the angiogenesis of renal cancer metastatic foci, and P-selectin on the platelet surface can mediate the adhesion of bladder cancer cells and vascular endothelial cells in the urinary tract, which is closely related to the metastatic pattern of urological tumors. This finding provides a potential basis for targeted anti-platelet therapy to inhibit urological tumor metastasis.

Hemoglobin was included in the multivariate Logistic regression as a continuous variable with an OR value of 0.977 (95%CI: 0.957–0.997), meaning that for every 1 g/L increase in hemoglobin level, the risk of urological tumor metastasis is reduced by 2.3%. Thus, a decrease in hemoglobin level is an independent risk factor for tumor metastasis, which may be related to the vicious cycle of urological tumor progression (16). On the one hand, chronic blood loss and bone marrow infiltration caused by tumor metastasis can directly reduce the hemoglobin level; on the other hand, the hypoxic microenvironment caused by anemia can activate the HIF-1α pathway, upregulating the expression of metastasis-related genes (such as Twist1) and accelerating the spread of tumor cells. This positive feedback mechanism of “hypoxia-metastasis” is more obvious in urological tumors with rich blood supply such as renal cancer and prostate cancer. The decrease of hemoglobin level can further aggravate the hypoxic microenvironment of the tumor tissue in the renal parenchyma and prostatic interstitium, and accelerate the tumor cell metastasis. This suggests that correcting anemia may become an important auxiliary strategy for improving the prognosis of urological tumor metastasis (17).

It should be noted that urological tumors include renal cancer, bladder cancer, prostate cancer and other types with significant heterogeneous biological characteristics. Bladder cancer is prone to pelvic lymph node metastasis, renal cancer has a high tendency of distant hematogenous metastasis, and prostate cancer is characterized by bone metastasis, and the inflammatory response and platelet activation status induced by different tumor types are also distinct. For example, bladder cancer tissue can induce a more intense local inflammatory response in the urinary tract mucosa, leading to a more significant increase in peripheral blood neutrophil count, while renal cancer, as a highly vascular tumor, is more likely to cause abnormal changes in platelet-related parameters due to tumor angiogenesis and microvascular damage. This study did not perform stratified analysis by tumor type due to the limited sample size of single tumor type after stratification, which may lead to potential bias of the general model in the prediction of metastasis risk for a single tumor type. However, the homogeneous distribution of tumor types between the training set and the validation set reduced the impact of tumor type heterogeneity on the model internal validation results to a certain extent. The combined prediction model constructed in this study is a general model for urological tumors, and its predictive efficiency and adaptability for a single tumor type need to be further verified by expanding the sample size of a single tumor type.

In this study, the AUC (0.891) of the random forest model was the highest among the three models. The reason why the random forest model was selected as the optimal model is that it has obvious advantages in comprehensive performance rather than a single AUC value. Its advantages stem from its adaptability to the characteristics of liquid biopsy data: First, it can effectively handle the non-linear relationship between biomarkers (such as the synergistic effect of neutrophil and platelet counts); second, by integrating multiple decision trees, it reduces the interference of single-indicator fluctuations (such as the influence of short-term infections on inflammatory indicators) on the prediction results; finally, the feature importance score quantifies the contribution of each indicator, providing a quantitative basis for clinical key monitoring of core indicators such as platelets and neutrophils. The clinical translational value of this model lies in its potential to guide specific management decisions in four scenarios. First, preoperative risk stratification for lymph node dissection (LND): For patients with urological tumors scheduled for radical surgery, a high predicted risk by our model (e.g., > 0.7) could prompt consideration of extended LND rather than standard LND, particularly in bladder and renal cancers where nodal micrometastases are common. Second, adjuvant therapy selection: High-risk patients may be candidates for adjuvant chemotherapy or, in future trials, targeted anti-inflammatory (e.g., COX-2 inhibitors) or anti-platelet agents (e.g., low-dose aspirin), which have shown potential in reducing metastasis risk. Third, surveillance intensity modification: High-risk patients could undergo more frequent postoperative imaging (e.g., every 3 months instead of the standard 6 months) to enable earlier detection of overt metastasis. Fourth, clinical trial enrichment: The model can be used to select high-risk patients for enrolling in clinical trials evaluating novel metastasis-prevention strategies. It is important to note that these clinical applications are currently proposed based on our retrospective data and require prospective validation before routine implementation. Nevertheless, for patients who cannot tolerate frequent imaging examinations (e.g., due to contrast allergy or renal impairment), this minimally invasive model offers a complementary risk assessment tool.

However, this study has some limitations: The single-center retrospective design may introduce selection bias. Furthermore, our study did not collect detailed information on the number or size of metastatic lesions (e.g., oligometastasis vs. polymetastasis). Therefore, we could not evaluate the model’s performance specifically for low-burden or early metastatic disease. The predictive value of our model for micrometastasis remains unproven, and we have tempered our claims accordingly throughout the manuscript. And the geographical homogeneity of the patient population may affect the external validity of the model; the liquid biopsy indicators do not cover emerging markers such as circulating tumor cells (CTC) and circulating tumor DNA (ctDNA), which may miss key predictive factors; the follow-up time is not clear, making it difficult to evaluate the long-term metastasis risk prediction efficacy of the model; the mechanism exploration relies on inferences from existing literature and lacks experimental verification. However, this study has some limitations: The single-center retrospective design may introduce selection bias, and the geographical homogeneity of the patient population may affect the external validity of the model; the liquid biopsy indicators do not cover emerging markers such as circulating tumor cells (CTC) and circulating tumor DNA (ctDNA), which may miss key predictive factors; the follow-up time is not clear, making it difficult to evaluate the long-term metastasis risk prediction efficacy of the model; the mechanism exploration relies on inferences from existing literature and lacks experimental verification. Additionally, external validation in independent multi-center cohorts is necessary to confirm the model’s generalizability. Finally, our study did not collect detailed information on the number or size of metastatic lesions (e.g., oligometastasis vs. polymetastasis). Therefore, we could not evaluate the model’s performance specifically for low-burden or early metastatic disease. The predictive value of our model for micrometastasis remains unproven.

In the future, multi-center prospective studies need to be carried out to expand the overall sample size and the sample size of single urological tumor type, and optimize the general prediction model by including emerging liquid biopsy indicators such as CTC and ctDNA and taking tumor type as an important confounding variable into the model construction. On this basis, stratified analysis by tumor type (renal cancer, bladder cancer, prostate cancer, etc.) will be performed to construct tumor type-specific prediction sub-models, so as to improve the stability and generalizability of the prediction model for different urological tumor types. In addition, combined with cell experiments and animal models of different urological tumor types, the molecular mechanism by which inflammatory-related and platelet parameters synergistically promote tumor metastasis in a type-specific manner should be analyzed; and the effectiveness of model-based individualized intervention strategies (such as targeted anti-inflammatory and anti-platelet treatment) in reducing the metastasis risk of different urological tumor types should be verified through randomized controlled trials (12, 18, 19).

In conclusion, inflammation-related and platelet parameters are key predictive factors for urological tumor metastasis. The random forest model constructed based on these liquid biopsy indicators has excellent predictive efficacy, and platelet count and neutrophil count can serve as core biomarkers for convenient clinical detection. This study provides new ideas for optimizing the diagnosis and treatment strategies of urological tumors from the perspective of “liquid biopsy-metastasis risk,” and is expected to improve patient prognosis through early intervention in the inflammation and platelet activation processes.

## Data Availability

The original contributions presented in this study are included in the article/Supplementary material, further inquiries can be directed to the corresponding author.
