# Supplementary material for: A prediction model for urological tumor metastasis using liquid biopsy-derived biomarkers
Source: Front Med (Lausanne). 2026 Jul 3;13:1718624. doi: 10.3389/fmed.2026.1718624 (PMC13377351; doi:10.3389/fmed.2026.1718624)
Supplement: Supplementary file 1 [file Table_1.docx]

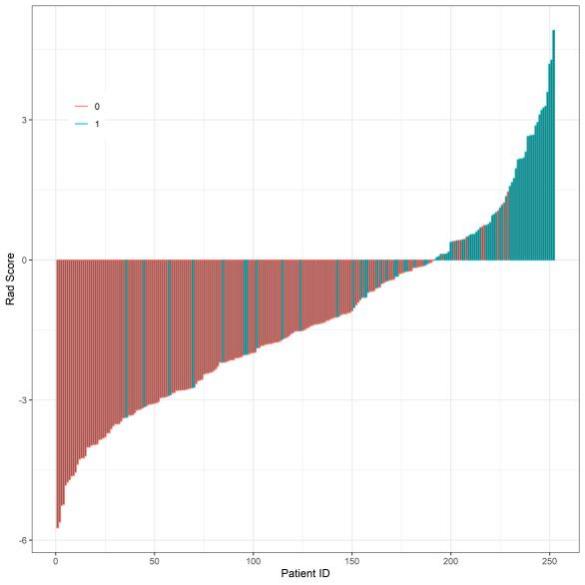


**Supplemental Figure 1.**LASSO score waterfall plot

**
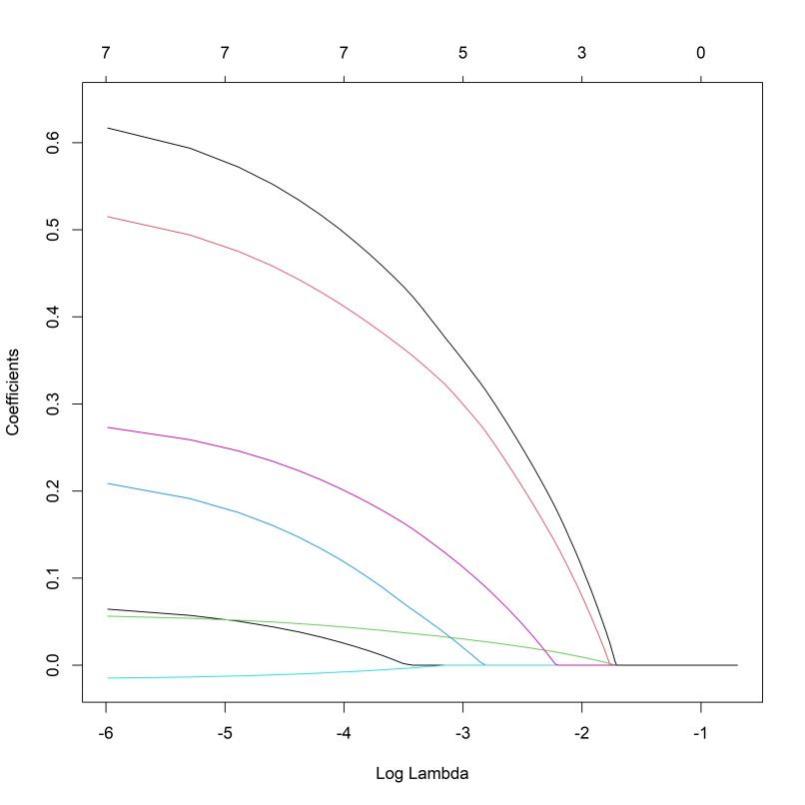
**

**Supplemental Figure 2.**LASSO regression analysis plot

**
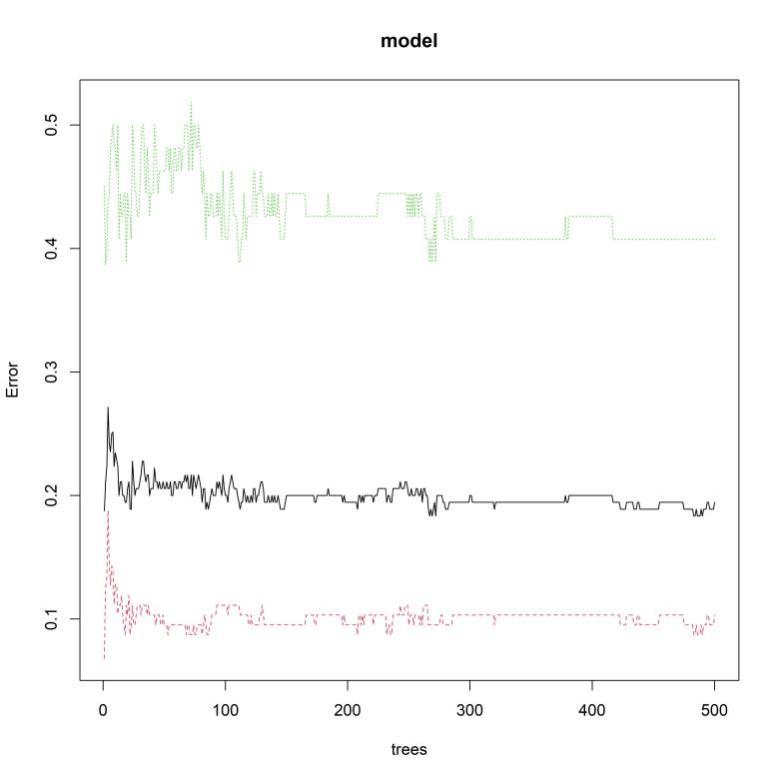
**

**Supplemental Figure 3.**Trend of the average out - of - bag estimation error rate with the number of decision trees


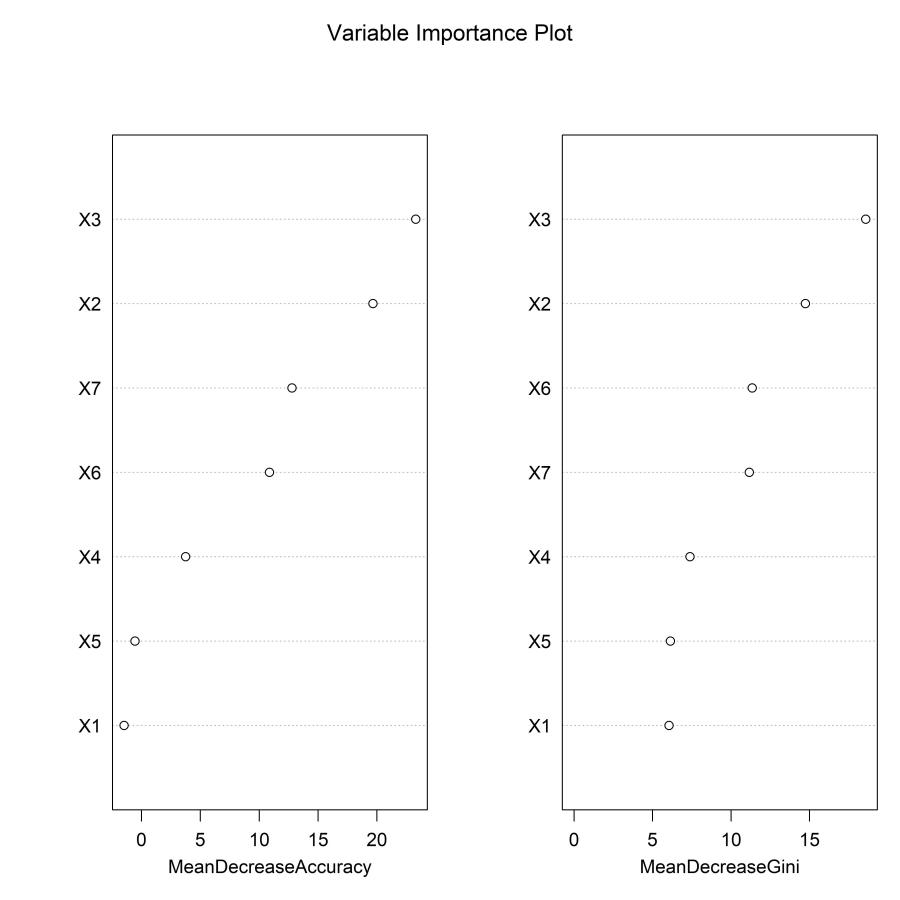


**Supplemental Figure 4.**Importance ranking of the random forest model (X1: CRP, X2: Neutrophil count, X3: PLT, X4: PDW, X5: Hemoglobin, X6: White blood cell count, X7: MPV)
