# Supplementary material for: A prediction model for urological tumor metastasis using liquid biopsy-derived biomarkers
Source: Front Med (Lausanne). 2026 Jul 3;13:1718624. doi: 10.3389/fmed.2026.1718624 (PMC13377351; doi:10.3389/fmed.2026.1718624)
Supplement: Supplementary file 2 [file Table_2.docx]

**Supplemental Table 1.** Variable assignment table

| Variable | Meaning | Assignment |
| --- | --- | --- |
| X1 | CRP | Continuous variable |
| X2 | Neutrophil count | Continuous variable |
| X3 | PLT | Continuous variable |
| X4 | PDW | Continuous variable |
| X5 | Hemoglobin | Continuous variable |
| X6 | White blood cell count | Continuous variable |
| X7 | MPV | Continuous variable |
| Y | Metastasis status | 1 = Metastasis group, 0 = Non - metastasis group |
