# Supplementary material for: A prediction model for urological tumor metastasis using liquid biopsy-derived biomarkers
Source: Front Med (Lausanne). 2026 Jul 3;13:1718624. doi: 10.3389/fmed.2026.1718624 (PMC13377351; doi:10.3389/fmed.2026.1718624)
Supplement: Supplementary file 3 [file Table_3.docx]

**Reproducible Prediction Pipeline for the Urological Tumor Metastasis Random Forest Mode**

Python code, model serialization (pickle), and instructions for external validation.

1. Model Overview

Algorithm: Random Forest Classifier

Target: Prediction of urological tumor metastasis (binary: 1 = metastasis, 0 = no metastasis)

Features (7 independent predictors identified by multivariate logistic regression):

C-reactive protein (CRP, mg/L)

Neutrophil count (×10⁹/L)

Platelet count (PLT, ×10⁹/L)

Platelet distribution width (PDW, %)

Hemoglobin (g/L)

White blood cell count (×10⁹/L)

Mean platelet volume (MPV, fL)

2. Model Hyperparameters (Final Tuned Model)

python

random_forest_params = {

'n_estimators': 500,

'max_depth': 10,

'min_samples_split': 5,

'min_samples_leaf': 2,

'random_state': 42,

'bootstrap': True,

'class_weight': 'balanced'

}

3. Complete Python Code for Model Training and Prediction

python

# -*- coding: utf-8 -*-

"""

Urological Tumor Metastasis Prediction Model - Random Forest

Based on 7 liquid biopsy biomarkers.

Author: Jiandong Qu et al.

Corresponding author: Xiaoli Huang (hxl19781004@126.com)

"""

import pandas as pd

import numpy as np

import pickle

from sklearn.ensemble import RandomForestClassifier

from sklearn.preprocessing import StandardScaler

from sklearn.metrics import roc_auc_score

# ================================

# 1. Data Preprocessing

# ================================

def preprocess_features(df, scaler=None, fit_scaler=False):

"""

Preprocess the 7 input features.

Parameters:

df : pandas.DataFrame with columns exactly:

['CRP', 'Neutrophil_count', 'PLT', 'PDW', 'Hemoglobin', 'WBC', 'MPV']

scaler : fitted StandardScaler (if fit_scaler=False)

fit_scaler : if True, fit a new scaler on the data

Returns:

X_scaled : numpy array of scaled features

scaler : fitted scaler (if fit_scaler=True)

"""

required_cols = ['CRP', 'Neutrophil_count', 'PLT', 'PDW', 'Hemoglobin', 'WBC', 'MPV']

X = df[required_cols].values

if fit_scaler:

scaler = StandardScaler()

X_scaled = scaler.fit_transform(X)

return X_scaled, scaler

else:

if scaler is None:

raise ValueError("When fit_scaler=False, a fitted scaler must be provided.")

X_scaled = scaler.transform(X)

return X_scaled

# ================================

# 2. Model Training (on training set of 252 patients)

# ================================

def train_model(X_train, y_train):

"""

Train the random forest model with predefined hyperparameters.

Parameters:

X_train : numpy array (n_samples, 7)

y_train : numpy array (n_samples,)

Returns:

model : trained RandomForestClassifier

"""

model = RandomForestClassifier(

n_estimators=500,

max_depth=10,

min_samples_split=5,

min_samples_leaf=2,

random_state=42,

bootstrap=True,

class_weight='balanced'

)

model.fit(X_train, y_train)

return model

# ================================

# 3. Prediction Function

# ================================

def predict_risk(model, scaler, input_data):

"""

Predict metastasis risk probability for new patients.

Parameters:

model : trained RandomForestClassifier

scaler : fitted StandardScaler

input_data : pandas.DataFrame with the 7 required columns (one or multiple rows)

Returns:

risk_scores : numpy array of probabilities (range 0-1)

"""

X_scaled = preprocess_features(input_data, scaler=scaler, fit_scaler=False)

risk_scores = model.predict_proba(X_scaled)[:, 1] # probability of metastasis

return risk_scores

# ================================

# 4. Example Usage (for external validation)

# ================================

if __name__ == "__main__":

# Example: Load your own external validation data

# Your data must contain the exact 7 columns with the same names and units.

# Units: CRP (mg/L), Neutrophil_count (×10⁹/L), PLT (×10⁹/L),

# PDW (%), Hemoglobin (g/L), WBC (×10⁹/L), MPV (fL)

# Replace this with actual data loading

# external_df = pd.read_csv("your_external_cohort.csv")

# For demonstration, create dummy data

sample_data = pd.DataFrame({

'CRP': [8.5, 6.2],

'Neutrophil_count': [6.5, 4.8],

'PLT': [260, 220],

'PDW': [17.0, 15.5],

'Hemoglobin': [125, 140],

'WBC': [7.8, 6.1],

'MPV': [11.2, 9.5]

})

# Load pre-trained model and scaler (provided in the same folder)

# The authors have serialized the model and scaler using pickle.

with open("rf_metastasis_model.pkl", "rb") as f:

model = pickle.load(f)

with open("scaler.pkl", "rb") as f:

scaler = pickle.load(f)

# Predict risk

risks = predict_risk(model, scaler, sample_data)

for i, risk in enumerate(risks):

print(f"Patient {i+1}: Metastasis risk probability = {risk:.3f}")

if risk > 0.7:

print(" -> High risk (consider extended LND, adjuvant therapy, intensified surveillance)")

elif risk > 0.3:

print(" -> intermediate risk")

else:

print(" -> Low risk")

4. Model Serialization Files (Provided as Pickle)

The trained model and scaler are serialized and provided in the supplementary ZIP folder:

rf_metastasis_model.pkl – The fitted RandomForestClassifier

scaler.pkl – The fitted StandardScaler (mean and std of each feature from the training set)

To load in Python:

python

import pickle

with open("rf_metastasis_model.pkl", "rb") as f:

model = pickle.load(f)

with open("scaler.pkl", "rb") as f:

scaler = pickle.load(f)

5. Instructions for External Validation

To validate our model on an independent cohort, please follow these steps:

Prepare your data as a CSV file with exactly the following columns (names case-sensitive):

CRP (mg/L, numeric)

Neutrophil_count (×10⁹/L, numeric)

PLT (×10⁹/L, numeric)

PDW (%, numeric)

Hemoglobin (g/L, numeric)

WBC (×10⁹/L, numeric)

MPV (fL, numeric)

Run the provided Python script (or use the code above) to load the model and scaler, then compute risk probabilities for each patient.

Compare predicted probabilities with actual metastasis status (defined per RECIST 1.1) in your cohort to calculate performance metrics (AUC, sensitivity, specificity, calibration).

Report your validation results and contact the corresponding author (Xiaoli Huang, hxl19781004@126.com) for collaboration or inquiries.

6. Expected Output Format

For each patient, the model outputs a risk score between 0 and 1, representing the probability of having urological tumor metastasis. Based on our training data distribution, we suggest the following risk categories (to be re-evaluated in external cohorts):

Risk Score Category Suggested Clinical Consideration

< 0.3 Low Standard surveillance

0.3 – 0.7 Intermediate Consider additional imaging

> 0.7 High Extended LND, adjuvant therapy, close follow-up

7. Dependencies

Python ≥ 3.7

pandas ≥ 1.0

numpy ≥ 1.18

scikit-learn ≥ 0.24

pickle (built-in)

Install required packages:

bash

pip install pandas numpy scikit-learn
